# Real-world evidence (RWE): A challenge for regulatory agencies discussion of the RWE conference with the network of the european medicine agencies, patients, and experts 

**DOI:** 10.3389/fphar.2022.969091

**Published:** 2022-07-26

**Authors:** Patrick Maison, Mahmoud Zureik, Virginie Hivert, Jesper Kjaer, Roman Hossein Khonsari, Gianluca Trifirõ, Christelle Ratignier-Carbonneil

**Affiliations:** ^1^ ANSM, Paris, France; ^2^ Faculté de Santé, Université Paris-Est Créteil, Créteil, France; ^3^ Epi-Phare, Saint Denis, France; ^4^ EURORDIS, Paris, France; ^5^ Data Analytics Centre, Danish Medicines Agency, Copenhagen, Denmark; ^6^ Health Data Hub, Paris, France; ^7^ Department of Diagnostics and Public Health, University of Verona, Verona, Italy

**Keywords:** real-world data (RWD), real-world evidence (RWE), big data, pharmacoepidemiology, pharmacovigilance, regulatory agencies, patient engagement, regulatory science

## 1 Introduction

Apart from its traditional use in the post-authorization phase for safety assessment, risk management, and life cycle benefit-risk evaluation (Flynn et al., 2022), the interest in Real-World Evidence (RWE) in the pre-authorization phase of medicines development is increasing exponentially (Li et al., 2021; Leufkens et al., 2022; Purpura et al., 2022). In this context, regulatory agencies should adapt their strategy (Heads of Medicines Agencies and European Medicines Agency, 2019a). RWE is defined as “the clinical evidence about the usage and potential benefits or risks of a medical product derived from analysis of Real-World Data (RWD)”; the latter includes “big data”, i.e., “extremely large datasets which may be complex, multi-dimensional, unstructured and heterogeneous, which are accumulating rapidly and which may be analyzed computationally to reveal patterns, trends, and associations” (Heads of Medicines Agencies and European Medicines Agency, 2019b).

RWE has a great potential not only to complement evidence generated through randomized controlled trials (RCTs) (Eichler et al., 2021) but also to provide valuable opportunities unavailable through RCTs (Chodankar, 2021). RCTs rightfully remain the fundamental method to establish the safety and efficacy of drugs for licensure (Slattery and Kurz, 2020). Nevertheless, the growing production and accessibility of digital health data support RWE to close the evidentiary gap between clinical research and real-world practice for better disease management (Corrigan-Curay et al., 2018; Lasky et al., 2021).

In March 2022, the Real World Evidence Conference involving the National Competent Authorities (NCAs) of European countries, patient representatives, and experts was held by the French medicine Agency (ANSM) in the framework of the French Presidency of the council of the European Union. The objectives of the Conference were to share recent initiatives of European member states in the field of RWE. Conference was concluded with a Roundtable, soliciting practical insights from patients, regulators, and experts addressing key problems facing regulatory agencies. The Roundtable specifically discussed specific challenges on how to ensure the rise in the competence of the agencies’ expertise and how to involve patients in this evolution.

Recently, the successful experience of using RWE for monitoring the safety and effectiveness of COVID-19 vaccines in the post-authorization represented an example of the interest of RWE to inform decision-making with rapidity, reactivity, agility, and transparency (Benkebil et al., 2021; Botton et al., 2022; Jabagi et al., 2022). RWE has been also successfully used for product approval or label expansion in situations where RCTs were unavailable, such as the cases of rare diseases lacking adequate treatment (Dreyer, 2018). Examples include the recent approval of Cufence for the treatment of Wilson’s disease in patients intolerant to D-Penicillamine therapy aged 5 years and older, based on a 12-months prospective continuation of a single group retrospective cohort study (Medicines Agency, 2022a). Another example is the conditional marketing authorization of the orphan medicine Zolgensma, gene therapy for spinal muscular atrophy, based on data from a longitudinal, multi-center, prospective natural history study (European Medicines Agency, 2022b).

The acceptability of RWE in the regulatory context faces numerous challenges. These encompass the understanding of the source and quality of RWD, the validity of new approaches and methods for processing, analyzing, and interpreting these data, which may for example incorporate algorithms or machine learning. A regulatory strategy is hence crucial to ascertain when and how RWE may be acceptable to ensure quality data for robust evaluation and inform decision-making over the life cycle of the drug (Heads of Medicines Agencies and European Medicines Agency, 2019b).

## 2 How to upskill the regulatory workforce to acquire the expertise needed to critically appraise the evidence generated by big data?

Regulatory agencies face several fresh challenges to keep pace with the data transformation for which the expertise is currently lacking. From the discussion, emerged two major axes to address the need for expertise in the use of emerging technologies and critical interpretation of analyses based on big data.

### 2.1 Expertise within regulatory agencies

The rapidly-evolving evidence landscape is forcing a shift in the way data are accessed, managed, analyzed, and utilized for decision-making (Heads of Medicines Agencies and European Medicines Agency, 2019b). Within this scope, the joint Big Data Task Force of EMA and the Heads of Medicines Agencies (HMA) proposed priority actions for the European medicines regulatory network to make the best use of big data to support evidence generation, innovation, and public health. These actions include delivering a sustainable platform to access and analyze data, enabling data discoverability, developing regulatory skills in big data, and building regulatory capability to analyze big data, among other priorities.

To date, a wide gap remains in the expertise and competencies needed to understand, analyze, interpret, and utilize big data at the level of regulatory agencies. A recent survey of the NCAs highlighted this gap: “What matter is not only access to data but access to education on how to handle the data. It is much riskier to have inexperienced researchers who have access to a large volume of data than having restricted access to data”.

As a first step toward solving this problem, regulatory agencies need to attract high-level scientific profiles, such as pharmacoepidemiologists, biostatisticians, data managers, and data scientists. The successful experience of the Health Data Hub (HDH) in France in this regard is acknowledged. The HDH’s public structure is attractive in nature to experts, given the value of working with the state and contributing to building long-term health data policy. Nevertheless, despite its public structure, the HDH has a certain level of freedom in recruiting and setting salaries, which enabled it to be competitive with private entities.

On another note, strengthening internal capacities in big data is needed. High-level training of current assessors on the characteristics of big data as well as the methodology and data processing inclusive of machine learning and artificial intelligence needs to be implemented.

In the long term, the partnership between academia and regulatory agencies, with different models of collaboration, is part of solving the knowledge gap: education should be customized to meet the needs of regulatory agencies. A successful example is illustrated by Epi-Phare (www.epi-phare.fr), a research group with permanent regulatory access to data from the French National Health Data System. Epi-Phare holds a strong partnership policy to develop pharmacoepidemiology in France by establishing expert committees. Efforts should also target undergraduate programs, since expertise in RWD is not only a technical issue, but also a cultural one, and future health professionals should become aware of RWD and the opportunities they provide early in their academic journey.

In conclusion, to meet immediate needs, building analytical expertise on RWD, suggested solutions included the recruitment of high-level expertise, training of current assessors to facilitate the dialogue with experts, and academic partnership.

### 2.2 Networking for data accessibility and quality

Second, partnerships need to be developed with expert networks in similar fields. Most importantly, there is a need for experience sharing by development methodologies to facilitate interagency exchanges at the European level.

Similarly, given the increasing competition between regulatory bodies and the private sector, establishing partnerships with sources of data is a key solution. For example, the HDH strives to build exclusive partnerships with potential sources of data. This is important, firstly to federate all data providers by helping to build local data warehouses in hospitals, and second to increase its data catalog. Furthermore, available data need to be strengthened to ensure that they are valid, reliable, and reproducible. Data also need to be easily and timely accessible and interoperable, while acknowledging the legal and regulatory issues to protect personal data.

Finally, regulatory bodies need to allocate funds to RWE. For example, in France, there are efforts to build a national policy for funding and federating the building of all hospital data warehouses in the country. Major authorities and the Ministry of Health are convinced that this is a national priority that should be put in place in the next few coming years. Epi-Phare’s efforts in this regard through funding doctoral and postdoctoral research, as well as pharmacoepidemiology centers, are highlighted.

### 2.3 How to involve patients in data-driven decision-making?

The momentum of incorporating patient perspectives at all stages of drug development is growing. This aims to create patient-focused healthcare solutions. Patients are actively contributing to the process of decision making, being in some cases are at its forefront, such as the case with EURORDIS. Exploring patients’ opinions, fears, and expectations from data inform policies regarding patient concerns and build public trust (Horgan et al., 2022).

The legitimacy of patient involvement was recognized during the COVID-19 pandemic. Amid the pandemic, the public and patients demonstrated increased health literacy and were sensitive to scientific evidence. Nowadays, citizens demand an appreciation of their healthcare data, as well as maximizing their value for themselves and other individuals. Second, patients should be aware of the potential of RWD and should also be consulted on the research questions that need to be addressed. For example, when patients and physicians were asked about the most important outcomes in a study of cystic fibrosis drugs, their views were misaligned. In contrast, the VALORE project involved patients through patient organizations in establishing priorities and obtaining their views on the analysis of big data. Third, the need for personal data security and confidentiality (Horgan et al., 2022), as well as credibility and transparency in dealing with RWD and generating RWE is constantly voiced out by patients. The National Competent Authorities (NCAs) need to discuss these issues with patients. The NCAs also should help develop the patients’ understanding of RWE. The survey conducted by EURORDIS on 2,000 patients with rare diseases regarding their feelings and preferences on sharing their health data revealed an extremely high willingness to share their data, regardless of disease severity or socio-demographic status (Courbier et al., 2019). Nevertheless, concerns were expressed related to the need for patients to be aware of how and by whom their data are utilized. Accordingly, measures are needed to protect patients, while ensuring data sharing. NCAs should maintain their responsibility toward patients and demonstrate their credibility, transparency, and scientific robustness (Horgan et al., 2022). For instance, the European platform DARWIN includes representatives of patients with rare diseases in its steering committee. Epi-Phare has no dedicated body that includes patients in the data analysis process, as this requires a high level of expertise. However, contact with patient organizations or associations is maintained, particularly for the dissemination of analysed data in published studies. Maintaining a strong relationship with patients can be illustrated by the experience of the HDH. First, the president of France Assos Santé, a cluster of patient associations, is part of the HDH’s top management. Second, the Patient and Citizen Office maintains relationships with the general public and patients through regular communications campaigns and specific documents. This aims to explore patients’ concerns, and familiarize them with the research that utilized their data. Another suggested approach to enhance patient trust is the publication of reports. Furthermore, to be able to understand the challenges and contribute meaningfully, patients need to be informed about the methods, strengths, and limitations of RWD. While the NCAs do not currently have the training capabilities or mandate to do this, they need to encourage patients to continue generating data and improve their understanding of RWE.

In conclusion, joining scientific robustness, credibility, and formation of partner patients are the cornerstone for patient involvement in the RWE process. Other suggested approaches to increase public trust in RWE include transparency and transnational access to RWE based on best practice guidance and applicable regulations.

## 3 Conclusion

The momentum is growing and significant steps are taken by regulatory medicine agencies to advance the use of RWE in decision making but challenges persist to accommodate change. Working smartly and collaboratively and embracing change are needed for agencies to evolve to deliver better data-driven decision-making and regulations for patients. Key solutions included establishing a framework for accessing and analyzing RWD through targeted recruitment of experts and training and developing the existing workforce, fostering multi-level collaborations with academia and data sources, and building a patient relationship through credible and transparent data analysis and dissemination of results.
