# Erratum: Real-world evidence (RWE): a challenge for regulatory agencies discussion of the RWE conference with the network of the European medicine agencies, patients, and experts

**DOI:** 10.3389/fphar.2025.1601645

**Published:** 2025-04-07

**Authors:** Frontiers Production Office

**Affiliations:** Frontiers Media SA, Lausanne, Switzerland

**Keywords:** real-world data (RWD), real-world evidence (RWE), big data, pharmacoepidemiology, pharmacovigilance, regulatory agencies, patient engagement, regulatory science

Due to a production error, in the article citation, the name of author Roman Hossein Khonsari was erroneously inserted as “Hossein Khonsari R” instead of “Khonsari RH”; additionally, in the copyright statement, it was mistakenly written as “Hossein Khonsari”, instead of “Khonsari”.

The publisher apologizes for this mistake.

The original version of this article has been updated.

